# Psoriasis Is Accompanied by Low Serum Levels of MG-H1, GOLD and MOLD: LC-Orbitrap-MS/MS Analysis of Chosen Glycation Products

**DOI:** 10.3390/molecules31091481

**Published:** 2026-04-29

**Authors:** Aleksandra Damasiewicz-Bodzek, Agnieszka Nowak, Maciej Maciejczyk, Magdalena Szumska, Sławomir Waligóra, Beata Pastuszka, Beata Janoszka

**Affiliations:** 1 Department of Chemistry, Faculty of Medical Sciences in Zabrze, Medical University of Silesia, 40-055 Katowice, Poland; aleksandra.bodzek@sum.edu.pl (A.D.-B.); maciekes7@gmail.com (M.M.); mszumska@sum.edu.pl (M.S.); swaligora@sum.edu.pl (S.W.); bjanoszka@sum.edu.pl (B.J.); 2 Research and Implementation Center Silesia LabMed, Medical University of Silesia, 40-752 Katowice, Poland; beata.gierat@sum.edu.pl

**Keywords:** psoriasis, AGEs, cross-linking, GOLD, MOLD, MG-H1

## Abstract

Glycation is a type of post-translational protein modification that affects antigen self-recognition, cell signaling, the proteasomal degradation of proteins, protein solubility, and the mechanical properties of tissues. Some glycation products are able to cross-link proteins. Serum concentrations of the cross-linking GOLD (glyoxal lysine dimer) and MOLD (methylglyoxal lysine dimer), and the non-cross-linking MG-H1 (methylglyoxal-derived hydroimidazolone isomer 1) in patients with psoriasis (*n* = 63) and in healthy controls (*n* = 35) were examined using the LC-Orbitrap-MS/MS (liquid chromatography–Orbitrap tandem mass spectrometry) technique. The following indices were assessed in the patients: BSA (body surface area), PASI (psoriasis area and severity index), and DLQI (Dermatology Life Quality Index). Serum concentrations of GOLD, MOLD, and MG-H1 were found to be significantly lower in psoriasis patients compared with healthy individuals. The concentrations of GOLD, MOLD, and MG-H1 did not correlate with the indices of disease activity and severity. These results may reflect the complexity of metabolic dysregulation accompanying immune-mediated inflammatory diseases such as psoriasis.

## 1. Introduction

Psoriasis is a chronic inflammatory skin disease affecting approximately 2–3% of the population, with equal prevalence in men and women. The essence of the disease is the accelerated proliferation and aberrant keratinization of epidermal cells, accompanied by inflammation in the dermis. Clinically, psoriasis most often manifests as the formation of well-demarcated erythematous plaques covered with silvery scales, which affect various, often extensive areas of the body [1,2]. The etiopathogenesis of the disease is a complex and still not fully understood interaction of genetic, immunological, hormonal, environmental, psychosomatic, and other factors. The central role is played by interleukin-23 (IL-23) and T helper cells (Th17). Dendritic cells and macrophages overproduce IL-23, which stimulates the differentiation and activity of Th17 cells. Activated Th17 lymphocytes release their effector cytokines, which in turn stimulate keratinocyte hyperproliferation and the production of proinflammatory mediators. This leads to a self-intensifying inflammatory process in psoriatic skin [1,2,3,4]. Psoriasis is frequently associated with metabolic comorbidities, including obesity, type 2 diabetes mellitus, cardiovascular disease, and metabolic syndrome [5].

The accelerated cellular metabolism in psoriasis contributes to oxidative stress. Oxidative environment promotes non-enzymatic protein glycation and subsequent formation of advanced glycation end products (AGEs) [5,6]. Glycation is a type of post-translational protein modification that affects antigen self-recognition [7], cell signaling [8], the proteasomal degradation of proteins [9], protein solubility [10], and the mechanical properties of tissues [11]. Some AGEs are able to cross-link protein chains and remain irreversibly bound to long-lived molecules [6,10]. AGEs activate the RAGE (receptor for advanced glycation end products). This interaction triggers pro-inflammatory signaling [5]. The profound impact of glycation on protein function and structure contributes to atherosclerosis, nephropathy, retinopathy, and neuropathy [11]. The AGEs-RAGE axis has also been shown to be involved in psoriasis etiopathogenesis through direct epidermal keratinocytes activation and the consequent amplification of psoriatic inflammation [12]. Several studies have documented elevated levels of total AGEs and chosen AGEs such as CML (N^6^-carboxymethyllysine), CEL (N^6^-carboxyethyllysine), and pentosidine, in both skin and serum of patients with psoriasis. In some of the studies, the levels of AGEs correlated with the disease severity [5,13,14]. These findings collectively suggest that the accumulation of AGEs may serve as a biomarker of psoriasis activity and may contribute to the pathogenesis of psoriasis-associated comorbidities.

**Figure 1:**
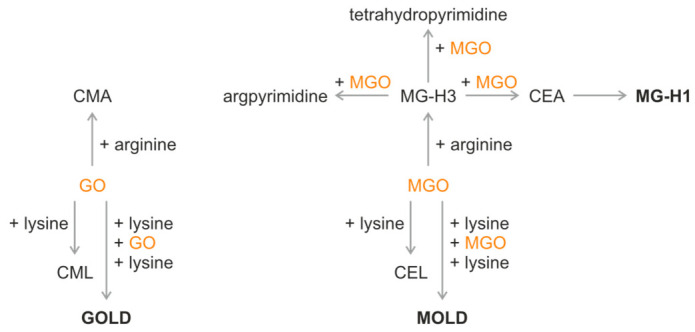
Simplified pathways utilizing amino acids and reactive α-dicarbonyl substrates in the formation of the chosen AGEs. CEA—N^7^-carboxyethylarginine; CEL—N^6^-carboxyethyllysine; CMA—N^7^-carboxymethylarginine; CML—N^6^-carboxymethyllysine; glyoxal—GO; GOLD—glyoxal lysine dimer; MG-H1—methylglyoxal-derived hydroimidazolone isomer 1; MG-H3—methylglyoxal-derived hydroimidazolone isomer 3; MGO—methylglyoxal; and MOLD—methylglyoxal lysine dimer.

Despite the growing evidence for elevated total AGEs in psoriasis, the behavior of the majority of glycation products has not been previously investigated in this disease. Arginine derivatives have not been assessed, and pentosidine was the sole cross-linking glycation product analyzed to this day. Examples of other protein cross-linking glycation products are GOLD (glyoxal lysine dimer) and MOLD (methylglyoxal lysine dimer). Among the non-cross-linking compounds are arginine derivatives such as CEA (N^7^-carboxyethylarginine), CMA (N^7^-carboxymethylarginine), MG-H1, MG-H2, and MG-H3 (methylglyoxal-derived hydroimidazolone isomers 1, 2, and 3). Substrates for their synthesis include reactive α-dicarbonyl compounds: GO (glyoxal) and MGO (methylglyoxal) [10,15]. Figure 1 depicts simplified pathways leading from the chosen α-dicarbonyl substrates to the AGEs.

Chronic inflammation is a characteristic feature of psoriasis, as explained previously. Inflammation and the production of AGEs are intertwined and self-amplifying processes [16]. Inflammation promotes the production of AGEs, hyperproliferation causes rapid amino acid turnover, and AGEs contribute to pro-inflammatory signaling and self-sensitization. Certain AGE subclasses, such as cross-linking AGEs, may additionally affect the properties of tissues negatively, decreasing their elasticity and contributing to psoriasis comorbidities. Thus, the aim of our study was to evaluate the serum concentrations of AGES, which have never been assessed in psoriasis patients before. The chosen glycation products included derivatives of amino acids and α-dicarbonyls: two lysine-derived cross-linking AGEs, GOLD and MOLD, as well as an arginine-derived non-cross-linking MG-H1 in patients suffering from psoriasis and in healthy controls, using a validated LC-Orbitrap-MS/MS (liquid chromatography–Orbitrap tandem mass spectrometry) method. Secondary objectives included an analysis of the relationships between these AGEs and clinical indices of psoriasis severity (BSA, PASI) and quality of life (DLQI).

## 2. Results

### 2.1. Determination of MG-H1, GOLD, and MOLD Using LC-Orbitrap-MS/MS

**Figure 2:**
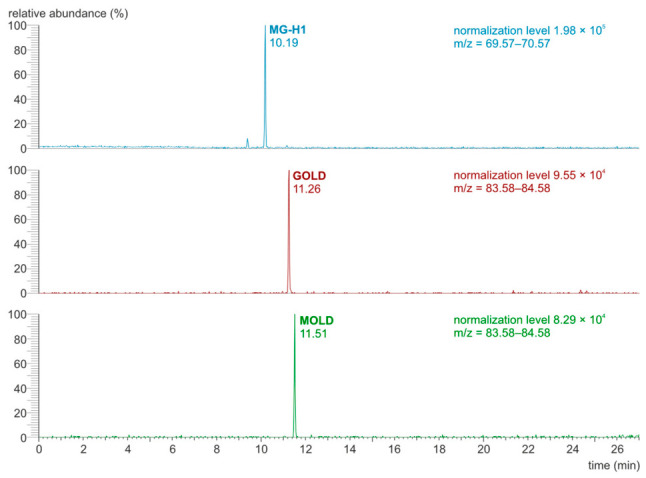
LC-Orbitrap-MS/MS chromatograms recorded in PRM mode for the AGE standard mixture (156.25 ng/mL, injection volume 1 μL).

**Figure 3:**
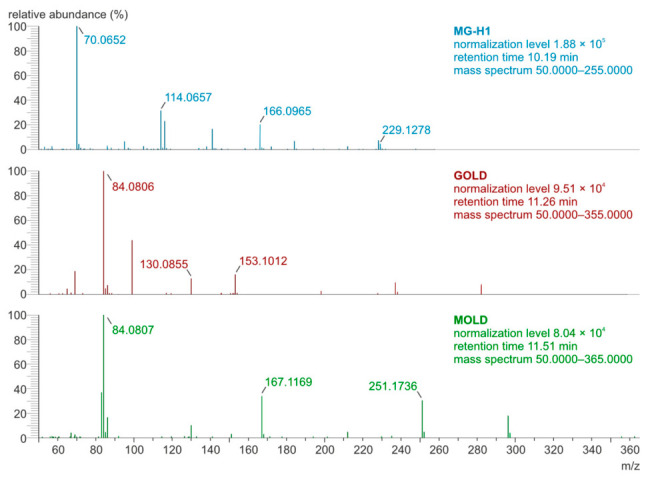
The mass spectra of MG-H1, GOLD, and MOLD standards recorded in PRM mode.

The original analytical procedure by Poojary et al. [17] was modified [18] to increase the resolution of chromatographic separation. The parallel reaction monitoring (PRM) mode was applied during the determination of the MG-H1, GOLD, and MOLD using the LC-Orbitrap-MS/MS technique. Figure 2 and Figure 3 show chromatograms and mass spectra of the MG-H1, GOLD, and MOLD standard mixture.

Determination of the optimal collision energies for generating product ions from protonated molecular precursor ions [M+H]^+^ involved modifications to the collision energy values to achieve maximum intensity of the product ions. Table 1 shows the selected optimal collision energies and masses of product ions formed from the precursor ions of the three AGEs that were determined in the blood serum.

The analyses of the MG-H1, GOLD, and MOLD were performed using linear calibration curves. The curves were prepared based on the results of 10 standard solutions analyses with concentrations ranging from 62.5 ng/mL to 1250 ng/mL. The curve for GOLD was linear, ranging from 62.5 ng/mL to 625 ng/mL. It was prepared on the basis of seven calibration points. Due to the low level of GOLD in the blood serum samples, the seven-point curve was used for the quantitative determination of this analyte in the tested biological material. The calibration curve equations and the correlation coefficients R for the determined AGEs are given in Table 2.

For all three selected AGEs, the LOD was determined based on the analysis of standard solutions of decreasing concentrations. The LOD was 20 ng/mL, and the LOQ was 60 ng/mL. These LOD and LOQ values correspond to 1 μg/mL and 3 μg/mL concentrations of these compounds in serum, respectively.

The recovery of the AGEs was calculated based on the results of a selected serum sample spiked with standards at the following concentrations: 6.25 μg/mL, 31.25 μg/mL, and 62.5 μg/mL. The recovery values and RSD% (relative standard deviation) shown in Table 3 are the average results obtained for the six serum samples spiked with standards at each of the three concentrations.

For the most spiked samples, the recovery values ranged from 87% to 120%. Only in the case of samples enriched with GOLD, at concentrations of 31.25 μg/mL and 62.5 μg/mL, were the obtained values excessively high, reaching approximately 150%. This result may have been caused by the relatively poor linearity (calibration correlation below 0.9) of the method observed for the higher GOLD concentrations (Table 2). The recovery rate for the enrichment of the serum sample with the GOLD standard at the lowest concentration of 6.25 ng/mL was 113%. This level of sample enrichment better corresponds to the GOLD concentrations determined in the tested serum samples. The GOLD concentrations were calculated using the calibration curve prepared in the lower concentration range of 62.5–625 ng/mL (Table 2), which exhibited better linearity (R = 0.982). Therefore, excessive recoveries at high sample enrichment levels should not affect the results of the GOLD assay in the serum.

The GOLD concentrations in the serum samples were low (ranging from 3.1 to 3.7 μg/mL); however, all the determined values exceeded the limit of quantification for this compound (3 μg/mL). All the tested serum samples from both study groups were subjected to hydrolysis and extraction under identical analytical conditions, and concentration calculations were performed using the same calibration curve. Therefore, systematic errors in the measurements should not significantly affect the validity of the comparison of study groups.

The relative matrix effect was estimated according to the method by Matuszewski et al. [19,20]. The method is based on the comparison between the slopes of standard calibration curves and the slopes of curves prepared for serum samples spiked with AGEs. All the concentration points applied in the standard curves were used in the calculation of the coefficient of variation CV (%). The CV values for the MOLD and MG-H1 slope lines were very similar and did not exceed 4–5%. The CV% for MOLD was 3.9%, and it was 3.3% for MG-H1. According to the authors of the papers mentioned above, these CV% values can be interpreted as the absence of significant sample matrix influence on quantification. In the case of GOLD, the CV% value was unfortunately very high (43%), which, in fact, confirmed the influence of the matrix on the GOLD analysis results. Since the calculation method we used involved serum samples spiked with standards that had undergone the entire analytical process, the obtained CV% and the associated relative matrix effect may have been significantly influenced by the sample preparation method (extraction and hydrolysis) rather than by the serum sample matrix itself. Furthermore, the limitations of the method used to assess the matrix effect should be considered. The calibration curve for the selected serum sample spiked with AGEs was prepared based on only three measurement points of three concentrations, which may have also contributed to the high matrix effect values for GOLD. The repeatability of the procedure was calculated as the RSD% of six values recorded over a single day. The results ranged from 0.93% to 1.42%. The intermediate precision expressed as the RSD% of the data recorded over five days was between 3.41% and 6.80%.

The injection volume was 1 µL for both standard solutions and the AGE extracts isolated from serum.

**Figure 4:**
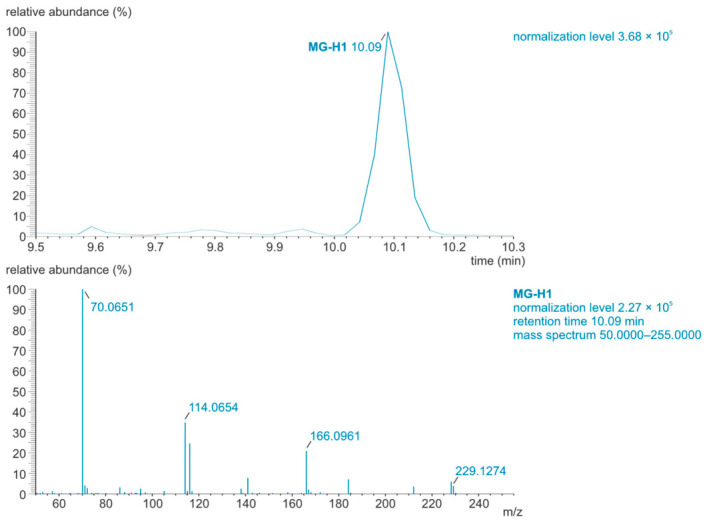
The representative LC-Orbitrap-MS/MS chromatogram (**top**) and mass spectrum (**bottom**) for the determination of MG-H1 in the control serum extract sample (recorded in PRM mode).

**Figure 5:**
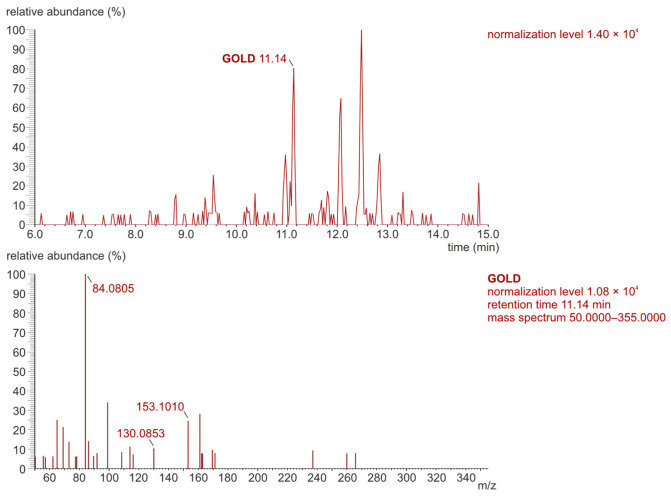
The representative LC-Orbitrap-MS/MS chromatogram (**top**) and mass spectrum (**bottom**) for the determination of the GOLD in the control serum extract sample (recorded in PRM mode).

**Figure 6:**
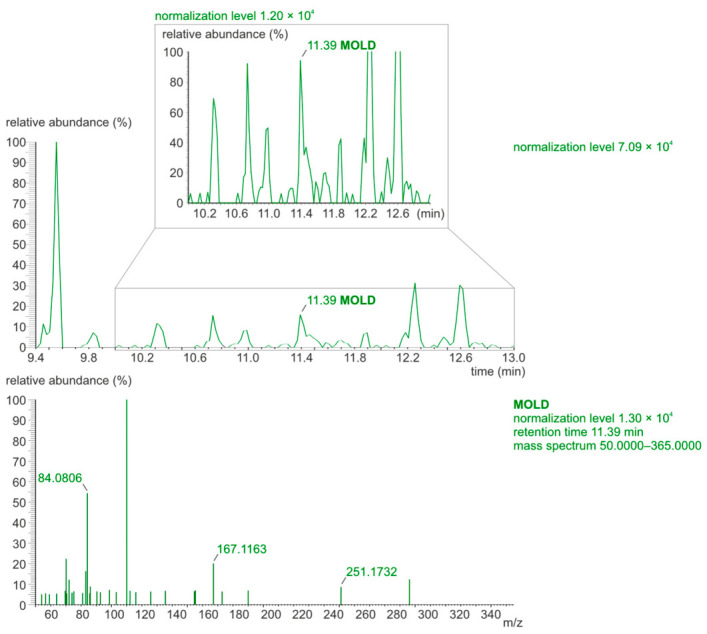
Representative LC-Orbitrap-MS/MS chromatogram (**top**) and mass spectrum (**bottom**) for the determination of the MOLD in the control serum extract sample (recorded in PRM mode). The top LC-Orbitrap-MS/MS chromatogram shows the MOLD’s retention time of 11.39 min and the zoomed-in neighboring peaks.

Representative chromatograms of the serum samples are shown in Figure 4, Figure 5 and Figure 6.

Between the chromatographic analyses of the consecutive serum samples, an analysis of acetonitrile was performed (as a background test). The exemplary chromatograms recorded for acetonitrile are included in the Supplementary Materials (Figures S1–S3). Acetonitrile analysis before and after each serum sample confirmed the absence of AGEs in the background. Only for MG-H1was there a small *m*/*z* signal observed in the mass spectrum recorded for a peak with a retention time closest to that of MG-H1, during the analysis of acetonitrile (Figure S1). The presence of this signal probably influenced the higher MG-H1 RSD value in the recovery experiment (Table 3).

Each sequence of the serum sample analyses ended with a duplicate analysis of standard solutions at a concentration of 625 ng/mL. The RSD for the GOLD and MOLD standard results obtained over a 2-month period was less than 6.5%, and the RSD was less than 9.5% for MG-H1. The retention times of the AGEs were also stable throughout the entire analytical process. The RSD% for the retention times of the MG-H1, GOLD, and MOLD, calculated for the standards injected at the beginning and the end of the sequence, was below 1%. No drifts in the retention times of the analyzed standards were observed.

### 2.2. Statistical Analysis of the Results

Quality of life and psoriasis severity indices for the patients involved in the study are presented in Table 4. The proportion of patients with different degrees of disease severity according to the Salgado-Boquete et al. [21] criteria is shown in Table 5.

**Figure 7:**
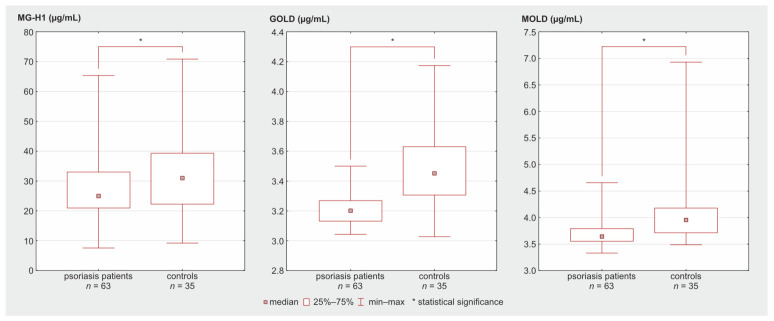
Quartiles and minimum–maximum range for the MG-H1, GOLD, and MOLD concentration in psoriasis patients and healthy controls.

The concentrations of all three of the compounds studied (MG-H1, GOLD, MOLD) did not differ significantly depending on gender and did not correlate with age, both in the study and control groups (Tables S1 and S2). The serum concentrations of MG-H1, GOLD, and MOLD were statistically significantly lower in psoriasis patients compared to the control group (Table 6, Figure 7). The effect size analysis has shown a medium effect for MG-H1, a large effect for MOLD, and a very large effect for GOLD (Table 6). After a cross-validation of the data, the psoriasis vs healthy control difference remained significant in the case of GOLD and MOLD. However, in the case of MG-H1, only half of the comparisons remained statistically significant.

A comparison of the mean concentrations of the studied compounds was also made between the group of patients with severe psoriasis and the group of patients with mild to moderate psoriasis. However, no statistically significant differences were found (Table 7).

In patients with psoriasis, the MG-H1 concentration in the serum correlated positively with the MOLD concentration (R = 0.40; *p* < 0.005) but not with the GOLD concentration (R = 0.12; *p* = 0.34). The MOLD concentration correlated positively with the GOLD concentration (R = 0.40; *p* < 0.005). In healthy individuals, there was only one correlation present—a positive correlation between the GOLD and MOLD concentrations (R = 0.61; *p* < 0.0005). In psoriatic patients, neither of the studied AGEs correlated with the indicators of disease severity (BSA, PASI), quality of life (DLQI), nor with duration of the disease (Table S3). The Bonferroni correction was used for the Type I error (false positives) check. The significant correlations remained significant after the correction.

## 3. Discussion

Glycation reaction, also known as glycoxidation, and the Maillard reaction, or nonenzymatic browning, begin with a carbonyl substrate and a free amine group. Among the carbonyl substrates are saccharides, including pentoses and hexoses (for example, glucose), and reactive dicarbonyl aldehydes, such as GO, MGO, and 3-deoxyglucosone. Amine group donors are free or protein-bound amino acids, primarily lysine and arginine. Glycation products are divided into early glycation products (EGPs), intermediate glycation products (IGPs), and advanced glycation end products (AGEs); however, this classification is not well defined due to the complexity of the process and the possibility of certain compounds acting as substrates, intermediates, and final products. Among glycation products, there are cross-linking AGEs, non-cross-linking AGEs, lysine derivatives, arginine derivatives, glyceraldehyde derivatives, heterocyclic compounds, and many more [22,23,24]. Despite their complex nature, AGEs have been proposed as markers of various conditions, and the usability of AGE inhibitors in treatment has been researched [25].

In previous studies, different parameters of glycation were assessed. Papagrigoraki et al. [14] reported an increased AGE accumulation in both the skin and serum of patients with severe psoriasis, while one of our previous studies [13] demonstrated the elevated circulating CML and CEL concentrations accompanied by the increased soluble RAGE levels. Two studies also showed that the mean concentration of the total AGEs in the serum of psoriasis patients was higher than in healthy subjects [26,27].

To our knowledge, this is the first study to simultaneously quantify the GOLD, MOLD, and MG-H1 in the serum of patients with psoriasis. The present study demonstrates that the serum concentrations of the cross-linking advanced glycation end products, GOLD and MOLD, were significantly lower in patients with plaque psoriasis compared with healthy controls. The serum MG-H1 levels were also reduced, but at a lower level of statistical significance compared to GOLD and MOLD. At first sight, the reduced concentrations of MG-H1, MOLD, and GOLD appear inconsistent with earlier reports demonstrating an increased total AGE accumulation in psoriasis [13,14,26,27]. However, the different AGE subclasses arise through partially separate biochemical pathways, have a distinct chemical nature, and may therefore reflect different aspects of the carbonyl and oxidative metabolism [23]. The present results are consistent with our previous observation of significantly lower serum MGO concentrations in psoriasis, together with inverse correlations between MGO and indices of disease activity and systemic inflammation [28]. Although the total circulating α-dicarbonyl pool was not directly quantified in the current study, these observations collectively suggest altered systemic carbonyl homeostasis in psoriasis. Interestingly, a comparable reduction in circulating α-dicarbonyl compounds has been reported in systemic lupus erythematosus [29], despite the generally high level of total AGEs in the blood [25], indicating that such metabolic alterations may represent a broader feature of immune-mediated inflammatory diseases.

In vivo synthesis of GOLD and MOLD is dependent on the availability of α-dicarbonyl substrates, GO and MGO, respectively. GO is a substrate for the GOLD formation, and MGO is a substrate for the MOLD and MG-H1 formation. Stoichiometrically, synthesis of GOLD requires two GO and two lysine molecules; synthesis of MOLD requires two MGO and two lysine molecules; and MG-H1 requires fewer substrates—one arginine molecule and one MGO molecule (Figure 1) [15,23]. Under the conditions of reduced α-dicarbonyl availability, reactions requiring multiple carbonyl substrates may theoretically be more susceptible to substrate limitation. Pathways leading to GOLD and MOLD might be more affected than the pathway leading to MG-H1. As a result, low serum concentrations of cross-linking AGEs could be observed in psoriasis patients. Although this concept has not been experimentally validated, it provides a hypothetical explanation for the more pronounced reduction observed for cross-linking AGEs compared with MG-H1.

**Figure 8:**
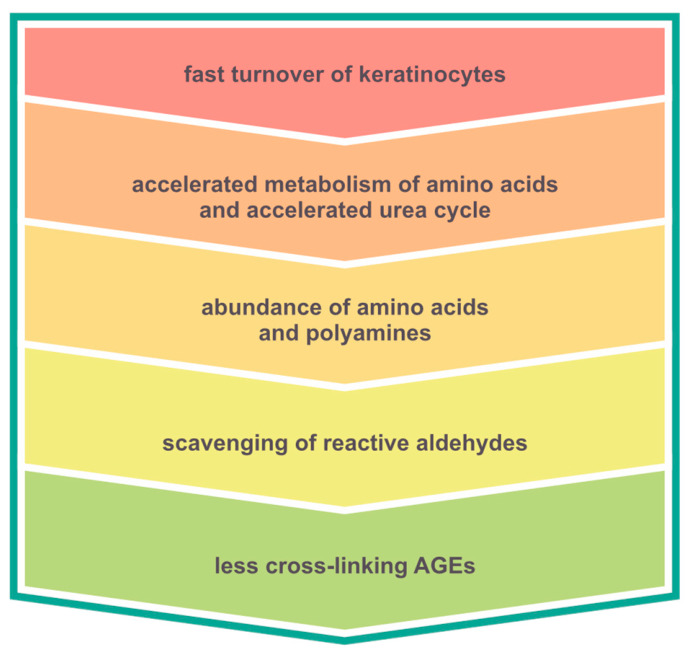
The possible causal connection between psoriasis pathogenesis and lower levels of cross-linking advanced glycation end products (AGEs) observed in the serum samples.

The shortage of α-dicarbonyl substrates may theoretically be attributed to the MGO-scavenging ability of polyamines because the experimental studies indicate that polyamines can interact with reactive carbonyl compounds and attenuate protein glycation reactions [30,31]. The increased concentration of polyamines—including spermine, spermidine, and putrescine—has been documented in psoriatic skin, blood, and urine [32,33,34,35,36]. This increase results from the accelerated metabolism of amino acids and the accelerated urea cycle caused by a fast turnover of keratinocytes. Gugliucci et al. have proven that polyamines protect proteins from glycation by reactive carbonyl compounds, including MGO [30]. For this reason, the concentrations of α-dicarbonyl-dependent AGEs may be decreased despite the general rise in AGEs observed in psoriasis. Although circulating polyamines were not measured in the present study, enhanced carbonyl scavenging mediated by polyamines represents one potential mechanism that could contribute to the reduced formation of cross-linking AGEs. The lesser extent of cross-linking in native proteins could be beneficial for their biological functions (Figure 8). On the other hand, polyamines may be metabolized to reactive cytotoxic aldehydes themselves [37]. Additionally, it is not known if a product of reaction between a polyamine and a reactive α-dicarbonyl may re-enter glycation pathways. Further studies should focus on the nature of polyamine and dicarbonyl reaction products because the overall biological consequences of altered carbonyl–amine interactions in psoriasis remain unknown.

Alternatively, the observed decrease in glycation products’ concentrations may stem from the choice of biological sample analyzed. The GOLD and MOLD are protein-bound molecules, and proteins are present in blood serum at a lower concentration than in the cells [38]. AGEs may remain retained in long-lived tissue proteins [10]. Therefore, analysis of skin lesions might provide a valuable insight into the distribution of glycation products in the tissues of patients affected by psoriasis.

The apparent coexistence of reduced cross-linking AGE concentrations and previously reported elevated CML and CEL concentrations in psoriasis may be explainable. CML and CEL are products of reactions involving GO and MGO, respectively. However, according to the Hodge pathway, the formation of AGEs, such as CML, is possible through oxidation and cleavage, without the involvement of α-dicarbonyl compounds [39]. Oxidative stress is a well-established feature of psoriasis pathophysiology [40]. Thus, CML and CEL formation may be independent of GO and MGO concentration fluctuations. Consequently, the oxidative mechanisms associated with chronic inflammation may sustain the formation of certain AGEs despite the reduced availability of circulating α-dicarbonyl precursors, whereas the formation of GOLD and MOLD appears to depend more directly on GO and MGO availability [15,23].

Importantly, the chosen cross-linking AGEs—MOLD and GOLD—were detected at low concentrations compared to MG-H1 (see Table 6 and Figure 2). According to Eble et al. [41], cross-linking AGEs are generally unstable under the acidic conditions applied during sample preparation, and MOLD and GOLD are exceptions. It is highly probable that other cross-linking compounds are present at high concentrations in the samples, but their detection is challenging due to their breakdown during analysis [41,42]. Model experiments suggest that under conditions of low α-dicarbonyl availability, the GOLD and MOLD formation is secondary to other cross-linking products, such as the GODIC (glyoxal-derived imidazolium cross-link) and the MODIC (methylglyoxal-derived imidazolium cross-link) [15]. While the exact steps of the GODIC and MODIC formation are unknown, it is proposed that synthesis of these molecules requires one α-dicarbonyl (either GO or MGO, respectively), one lysine, and one arginine molecule [43]. The significance of MOLD and GOLD in biological samples may be overestimated [10]. Among methylglyoxal-derived hydroimidazolone isomers, MG-H1 is the most thermodynamically stable and abundantly formed in vivo [44]. Recoveries reported after acid hydrolysis in food samples are 67–129% for MOLD, 61–111% for GOLD, and 75–106% for MG-H1. General recoveries achieved after various types of hydrolysis are 67–129% for MOLD, 58–118.6% for GOLD, and 70–123% for MG-H1; however, many studies do not include recovery data [45].

In the current study, no significant correlations were identified between analyzed AGE concentrations and clinical indices of disease severity (PASI, BSA) or quality of life impairment (DLQI). Within the psoriasis group, positive correlations were observed between MG-H1 and MOLD as well as between MOLD and GOLD, whereas in healthy controls, a significant correlation was only detected between GOLD and MOLD. These results additionally suggest that MG-H1, GOLD, and MOLD cannot be considered biomarkers of disease course and severity, despite the clear link between glycation and psoriasis documented in the previous studies.

Several limitations of our study should be noted. All patients were examined during hospitalization for disease exacerbation prior to therapy initiation; therefore, the potential effects of treatment on circulating cross-linking AGEs could not be evaluated. Exclusion of major metabolic comorbidities increases the study group homogeneity but may limit the possibility of inference to the broader psoriasis population. Knowing the importance of metabolic disorders in the formation of glycation products, our aim was to eliminate confounding factors to the best of our ability. We are, however, aware that the psoriasis patient population is more heterogeneous than our study population. Circulating polyamines and α-dicarbonyl intermediates were not measured, and the proposed interpretation should therefore be regarded as hypothetical. Future studies should concurrently investigate indices of disease severity and inflammation, α-dicarbonyl levels, polyamine concentration, and longitudinal changes in AGEs in relation to treatment response and disease activity. The relationship of glycation parameters and additional data, such as body mass index (BMI) and lifestyle factors (diet, smoking, and alcohol use), could be assessed to better characterize the studied group. Expansion of analysis to include additional biological material may further clarify whether reduced serum concentrations reflect altered tissue distribution, enhanced retention, or diminished systemic formation.

The strength of our study is the application of a high-resolution, structurally specific LC-Orbitrap-MS/MS method in PRM mode to quantify the GOLD, MOLD, and MG-H1 in human serum. This method enables the separation and determination of structurally similar compounds at very low concentrations compared with immunoenzymatic or fluorescence-based techniques previously applied in psoriasis studies [13,14,17].

Despite applying such a sensitive and selective chromatographic technique, we have encountered challenges during the determination of GOLD in the serum samples. Unfortunately, the recovery values for GOLD were quite high, which may suggest the significant effect of the matrix and sample pretreatment on this specific glycation product concentration. As a part of our further research, we plan to optimize the method for sample preparation and to examine the influence of the matrix on the results of AGE determination more closely. The use of isotope-labeled internal standards may greatly decrease these matrix effects on the assay results, particularly for AGEs that are present in the serum at low concentrations.

## 4. Materials and Methods

### 4.1. Chemicals and Reagents

The analytical standards MG-H1 (96.6%, mixture of diastereoisomers), GOLD (98.9%, acetate salt), and MOLD (99.8%, acetate salt) were purchased from Iris Biotech GmbH (Marktredwitz, Germany). The hydrochloric acid (37%, analytical grade) was obtained from Chemland (Katowice, Poland). The acetonitrile (99.97%, LiChrosolv^®^ hypergrade, suitable for LC-MS), LC-MS-grade water (≤1 µS × cm^−1^, LiChrosolv^®^ hypergrade), and perfluoropropionic acid (99%, PFPA) were supplied by Sigma-Aldrich (Darmstadt, Germany). Ultrapure water (18.2 MΩ × cm) was produced using a Polwater DL-2 deionizer (Labopol-Polwater, Kraków, Poland). Pierce™ LTQ Velos ESI Positive Ion Calibration Solution was purchased from Thermo Fisher Scientific (Rockford, IL, USA) and used for mass spectrometer tuning and calibration. High-purity nitrogen (≥99.99%) was purchased from SIAD Poland Sp. z o.o. (Ruda Śląska, Poland).

### 4.2. Study Population and Sample Collection

The Local Bioethical Commission of the Medical University of Silesia in Katowice approved the study design (approval number PCN/CBN/0052/KB1/63/I/22, granted on 20 September 2022). Participants provided informed, written consent, and their participation was voluntary.

The study included 63 plaque psoriasis patients (40 men and 23 women, mean age 53.4 ± 14.6 years) who received medical attention in a dermatological ward due to disease exacerbation. It was the first clinical manifestation of psoriasis in the case of two patients; the remainder of the group had already experienced clinical manifestations in the past. The mean time since psoriasis onset was 17.6 ± 15.6 years (minimum 1 month, maximum 60 years). Any comorbidity was considered an exclusion criterion. The patients underwent disease severity and quality of life assessment with the use of the following indices: the body surface area (BSA) score, the psoriasis area and severity index (PASI), and the Dermatology Life Quality Index (DLQI). Patients were always qualified and examined by the same dermatologist. Venous blood was collected after an overnight fast but before the initiation of treatment. The patients had not received any treatment except topical emollients for at least ten days prior to sampling.

The control group included 35 healthy individuals (15 men and 20 women) of comparable age (47.5 ± 13.8 years, *p* > 0.05). A family history of psoriasis was considered an exclusion criterion. Subjects in both groups, psoriasis and control, were of Caucasian ethnicity. The venous blood was collected after an overnight fast.

The collected blood samples were allowed to clot and then centrifuged. The obtained serum was stored frozen at −80 °C until analysis.

### 4.3. Stock Solutions and Calibration Standards for LC-MS/MS Analysis

Individual 10 mg/mL stock solutions of MG-H1, GOLD, and MOLD were prepared by dissolving each compound in LC-MS-grade water. A mixed working solution containing all three analytes at a 10 µg/mL concentration was prepared by combining appropriate volumes of the individual stock solutions and diluting with LC-MS-grade water. The calibration standards were freshly prepared on the day of analysis by serial dilution of the mixed working solution in LC-MS-grade water, covering the concentration range of 62.5–1250 ng/mL.

### 4.4. Serum Samples Preparation

Serum samples were thawed at a controlled room temperature (22 ± 2 °C) and gently mixed by inversion. For acid hydrolysis, an aliquot of 20 µL of serum was transferred into 2 mL amber glass vials. Subsequently, 100 µL of 6.6 M hydrochloric acid prepared in LC-MS-grade deionized water was added to each vial. The mixtures were vortexed for 10 s at 3000 rpm to ensure homogeneity (VELP Scientifica vortex, Usmate Velate, Italy). To minimize oxidative degradation of the analytes, the headspace of each vial was purged in EVA EC-S evaporator (VLM GmbH, Bielefeld, Germany) with high-purity nitrogen at a flow rate of 50 mL/min for 1 min. The vials were then tightly sealed with screw caps equipped with polytetrafluoroethylene-lined septa and incubated in a temperature-controlled oven SLN 53 EKO (POL-EKO^®^, Wodzisław Śląski, Poland) at 90 ± 1 °C for 24 h. Following hydrolysis, the samples were evaporated to dryness under a gentle stream of nitrogen (20 mL/min) at 40 °C, typically requiring approximately 25 min. The resulting residues were reconstituted in 1.0 mL of LC-MS-grade water, vortexed at 3000 rpm for 1 min, and subjected to ultrasonication in a temperature-stabilized ultrasonic bath (Sonic 6, Polsonic, Warsaw, Poland; 40 kHz, 30 min, 25 ± 2 °C) to facilitate complete dissolution and desorption. Clarification of the extracts was achieved by centrifugation at 13,500 rpm for 15 min (MPW-55 centrifuge, MPW MED. INSTRUMENTS, Warsaw, Poland). The supernatants were collected and passed through 0.2 µm polyethersulfone (PES) syringe filters. Filtered extracts were stored at 4 °C until analysis. Two independent extractions were prepared from each serum sample. An injection volume of 1 µL was used for LC-MS/MS analysis.

### 4.5. Conditions of Chromatographic Separation and MS

To determine the content of the chosen AGEs in the samples, we used a procedure developed by Poojary et al. [17], which we modified and applied to determine the AGE content in the infant formula in our previous study [18]. The conditions of chromatographic separation and MS detection are presented in Table 8, Table 9 and Table 10. In order to ensure the repeatability and accuracy of measurements, the mass spectrometer was calibrated once a week using a positive ion calibration solution.

The optimized collision energies for each analyte are listed in Table 1.

### 4.6. Basic Validation Parameters

The validation of the method for determining AGEs involved assessing their linearity, limit of detection (LOD), limit of quantitation (LOQ), repeatability, intermediate precision, recovery rates, and relative matrix effect.

Linearity was determined using the solutions listed in Section 4.3. In order to determine the LOD, standard solutions of decreasing concentrations were analyzed. The LOD was defined as the concentration at which the ratio of the analyte signal area to the noise signal area was 3 on the chromatogram. The LOQ was set at three times the LOD. To determine the recovery rates, the serum sample with the lowest AGE content was selected and then spiked with standards. The spiking levels were as follows: 6.25 μg/mL, 31.25 μg/mL, and 62.5 μg/mL. Enriched samples underwent the AGE fraction isolation procedure described in Section 4.4, and then the concentration of analytes was determined using the LC-Orbitrap-MS/MS technique in PRM mode. Recovery (in %) was calculated using the formula:

$$recovery=\frac{c_{1}-c_{2}}{c}\cdot 100\%$$

where c is the concentration (µg/mL) of standard added to the serum sample, c_1_ is the concentration of the analyte in the standard-enriched serum (µg/mL), and c_2_ is the analyte concentration in the serum (µg/mL) without the addition of the standard.

The experiment conducted to determine AGE recovery rates was also used to estimate the relative matrix effect, in accordance with one of the methods proposed by Matuszewski et al. [19,20]. The method we chose takes into account the slopes of the calibration curves for standards and the slopes of the curves prepared for the serum samples spiked with AGEs at three concentrations. These samples underwent the full analytical procedure, meaning that AGEs were added to them prior to hydrolysis and extraction. The CV% calculated for the standard curve slopes and for the spiked sera curve slopes served as their precision indicator.

The repeatability of the AGE determination procedure was calculated as the RSD% of values recorded over a single day, based on six consecutive analyses. The intermediate precision of the data was calculated using data recorded over five days.

### 4.7. Statistics

The results were presented using basic parameters of descriptive statistics. The consistency of the distribution of variables with the normal distribution was checked using the Shapiro–Wilk test. Kolmogorov–Smirnov and Mann–Whitney U tests were used to compare the psoriasis and healthy groups. Spearman’s rank correlation test was used to analyze the correlations. For significant differences, the effect size was also examined (Cohen’s d). For multiple correlation analysis, the Bonferroni correction was used. Additionally, the study groups were divided into sub-cohorts for the cross-validation of data. A value of *p* < 0.05 was considered statistically significant. Statistical analysis was performed using the TIBCO Statistica version 13.3 (TIBCO Software Inc., Palo Alto, CA, USA).

## 5. Conclusions

The concentrations of the cross-linking AGEs MOLD and GOLD, as well as non-cross-linking MG-H1, are decreased in psoriasis, despite the substantial evidence of exacerbated oxidative stress and chronic inflammation in this disease. The lack of correlation between these AGE concentrations and indices of disease activity and severity may reflect the complexity of metabolic dysregulation accompanying immune-mediated inflammatory diseases such as psoriasis. In conclusion, MOLD, GOLD, and MG-H1 are not linear biomarkers of psoriasis severity in the comorbidity-free cohort.

## Figures and Tables

**Table 1:** The parameters of PRM mode during the LC-Orbitrap-MS/MS AGE determination.

Analyzed Compound	Retention Time (min)	Precursor Ion → Product Ion (*m*/*z*)	Collision Energy [eV]
MG-H1	10.19	229.1278 → 70.0652	20
GOLD	11.26	327.2000 → 84.0806	35
MOLD	11.51	341.2000 → 84.0806	37

**Table 2:** The calibration parameters for the AGEs determined by LC-Orbitrap-MS/MS in PRM mode.

Analyzed Compound	Calibration Curve Equation	R	Calibration Curve Concentration Range [ng/mL]
MG-H1	y = 4976.06x − 218.614	R = 0.986	62.5–1250
GOLD	y = 2469.08x − 144.467	R = 0.886	62.5–1250
GOLD ^1^	y = 1622.8x − 515.75	R = 0.982	62.5–625
MOLD	y = 2270.49x − 144.723	R = 0.996	62.5–1250

^1^ curve used for the quantitative determination of the GOLD in serum samples.

**Table 3:** The recovery values of AGEs from serum samples enriched with standards.

Analyzed Compound	Recovery (%) for Spiking Level (µg/mL of Serum); *n* = 6					
	6.25 µg/mL	RSD (%)	31.25 µg/mL	RSD (%)	62.5 µg/mL	RSD (%)
MG-H1	115.3	29.9	87.0	18.3	102.5	16.1
GOLD	113.4	3.7	149.3	2.9	155.1	5.2
MOLD	97.0	6.7	119.8	4.0	119.6	3.7

**Table 4:** The mean, median, and minimum–maximum range for the BSA, PASI, and DLQI in the patients with psoriasis.

Clinical Parameters	Mean ± SD	Median (Min–Max Range)
Body Surface Area (BSA) [%]	22.3 ± 17.8	19.9 (1.0–71.0)
Psoriasis Area and Severity Index (PASI)	13.1 ± 9.3	11.2 (2.0–43.8)
Dermatology Life Quality Index (DLQI)	13.5 ± 6.2	9.9 (2.0–26.0)

SD—standard deviation.

**Table 5:** The percentage of patients involved in the study according to the psoriasis severity criteria proposed by Salgado-Boquete et al. [21].

% of Patients	Psoriasis Type	Classification Criteria
56.0%	severe	PASI ≥ 11 and BSA ≥ 10%, any DLQI
42.5%	moderate	PASI ≥ 11 or BSA ≥ 10%, DLQI ≥ 5
1.5%	mild	PASI ≤ 3 or BSA ≤ 5%, DLQI < 5

**Table 6:** The serum concentrations of MG-H1, GOLD, and MOLD in psoriatic patients and healthy controls.

Compound	Mean Concentration ± SD (95% Confidence Interval) (μg/mL)		*p* Value	Cohen’s d
	Psoriasis Patients (*n* = 63)	Controls (*n* = 35)		
MG-H1	27.27 ± 10.71 (24.57–29.96)	33.37 ± 14.41 (28.75–42.47)	*p* < 0.05	−0.59
GOLD	3.21 ± 0.11 (3.19–3.24)	3.48 ± 0.25 (3.39–3.57)	*p* < 0.000001	−1.56
MOLD	3.71 ± 0.25 (3.65–3.78)	4.15 ± 0.73 (3.88–4.41)	*p* < 0.00005	−0.93

SD—standard deviation.

**Table 7:** The concentrations of examined compounds based on psoriasis severity.

Compound	Mean Concentration ± SD (μg/mL)		
	Severe Psoriasis (*n* = 35)	Mild to Moderate Psoriasis (*n* = 28)	*p* Value
MG-H1	28.23 ± 12.38	25.33 ± 7.44	*p* > 0.1
GOLD	3.21 ± 0.10	3.22 ± 0.11	*p* > 0.5
MOLD	3.73 ± 0.28	3.68 ± 0.21	*p* > 0.5

**Table 8:** Details of the equipment used for AGE determination.

Compartment	Details	Manufacturer
Column	Phenomenex BioZen 1.7 µm Peptide XB-C18 LC column (150 × 2.1 mm × 1.7 μm)	Phenomenex, Værløse, Denmark
Liquid Chromatograph	Vanquish Thermo Scientific, consisting of: Double split sampler set at 1 μL injection volume and 10 °C; Column thermostat compartment set at 40 °C; Double pump set at 0.250 mL/min flow rate, with a multistep gradient of A: 5 mM PFPA in water and B: 5 mM PFPA in acetonitrile.	Thermo Fisher Scientific Inc., Waltham, MA, USA
Mass Spectrometer	Thermo Scientific OrbiTrap Q Exactive with a heated electrospray ionization (HESI) source	Thermo Fisher Scientific Inc., Waltham, MA, USA
Nitrogen Source	NiGen LCMS 40-1 generator	Claind Brezza, Tremezzina, Italy
Software	Thermo Xcalibur LC Devices 3.2 Robust Thermo Xcalibur version 4.4.16.14	Thermo Fisher Scientific Inc., Waltham, MA, USA

**Table 9:** The multistep gradient applied during chromatographic separation.

Analysis Time	Composition of the Mobile Phase
0–2 min	100% A ^1^
2.01–5 min	80% A, 20% B ^2^
5.01–7 min	75% A, 25% B
7.01–9 min	70% A, 30% B
9.01–16 min	65% A, 35% B
16.01–20 min	60% A, 40% B
20.01–28 min	100% B
28.01–40 min	100% A

^1^ A: 5 mM PFPA in water; ^2^ B: 5 mM PFPA in acetonitrile.

**Table 10:** Orbitrap MS/MS detection parameters.

Parameter	Value
Ion Spray Voltage	3.5 kV
Ion Transfer Capillary Temperature	320 °C
Sheath Gas Flow Rate	30 arbitrary units
Auxiliary Gas Flow Rate	4 arbitrary units
Sweep Gas Flow Rate	0 arbitrary units
S-Lens Radio Frequency Level	50%
*m*/*z* Scan Range	70–1000 *m*/*z*
*m*/*z* Scan Resolution	17,500 full-width at half maximum (FWHM)
Automatic Gain Control (AGC) Target	2 × 10^5^
Maximum Injection Time	64 ms
Quantification Mode	parallel reaction monitoring (PRM)

## Data Availability

The data is contained within the article or Supplementary Materials.

## Supplementary Materials

The following supporting information can be downloaded at https://www.mdpi.com/article/10.3390/molecules31091481/s1. Figure S1: Fragments of the LC-Orbitrap-MS/MS chromatogram for the determination of MG-H1 in serum extract sample and acetonitrile analyzed prior to the serum sample; Mass spectrum for the signal near the MG-H1 retention time recorded in acetonitrile sample title; Figure S2: Fragments of the LC-Orbitrap-MS/MS chromatogram for the determination of GOLD in serum extract sample and acetonitrile analyzed prior to the serum sample; Mass spectrum for signals near the GOLD retention time recorded in acetonitrile sample title; Figure S3: Fragments of the LC-Orbitrap-MS/MS chromatogram for the determination of MOLD in serum extract sample and acetonitrile analyzed prior to the serum sample; Mass spectrum for the signal near the MOLD retention time recorded in acetonitrile sample title. Table S1: serum concentrations of the examined compounds stratified by sex; Table S2: correlation analysis between concentrations of the examined compounds and age of the study participants; Table S3: correlation analysis between concentrations of the examined compounds and indicators of disease severity (BSA, PASI), quality of life (DLQI), and duration of the disease.

## Abbreviations

The following abbreviations are used in this manuscript:

AGC	Automatic gain control
AGEs	Advanced glycation end products
BMI	Body mass index
BSA	Body surface area
CEA	N^7^-carboxyethylarginine
CEL	N^6^-carboxyethyllysine
CMA	N^7^-carboxymethylarginine
CML	N^6^-carboxymethyllysine
CV%	Coefficient of variation
3-DG	3-deoxyglucosone
DLQI	Dermatology Life Quality Index
DOLD	3-deoxyglucosone lysine dimer
EGPs	Early glycation products
FWHM	Full width at half maximum
GO	Glyoxal
GODIC	Glyoxal-derived imidazolium cross-link
GOLD	Glyoxal lysine dimer
HESI	Heated electrospray ionization
IGPs	Intermediate glycation products
IL-23	Interleukin 23
LC	Liquid chromatography
LC-MS/MS	Liquid chromatography–tandem mass spectrometry
LOD	Limit of detection
LOQ	Limit of quantification
MG-H1	Methylglyoxal-derived hydroimidazolone 1
MG-H2	Methylglyoxal-derived hydroimidazolone 2
MG-H3	Methylglyoxal-derived hydroimidazolone 3
MGO	Methylglyoxal
MODIC	Methylglyoxal-derived imidazolium cross-link
MOLD	Methylglyoxal lysine dimer
MS	Mass spectrometry
MS/MS	Tandem mass spectrometry
PASI	Psoriasis area and severity index
PES	Polyethersulfone
PFPA	Perfluoropropionic acid
PRM	Parallel reaction monitoring
RAGE	Receptor for advanced glycation end products
RSD%	Relative standard deviation
