# Supplementary material for: Psoriasis Is Accompanied by Low Serum Levels of MG-H1, GOLD and MOLD: LC-Orbitrap-MS/MS Analysis of Chosen Glycation Products
Source: Molecules. 2026 Apr 29;31(9):1481. doi: 10.3390/molecules31091481 (PMC13165278; doi:10.3390/molecules31091481)
Supplement: Supplementary file 1 [file molecules-31-01481-s001.zip › molecules-4202571-supplementary.pdf]

Table S1. Serum concentrations of the examined compounds stratified by sex.

**Compound**	**Mean Concentration ± SD (µg/mL)**		
	**Psoriasis Patients (n = 63)**		
	**Men (n = 40)**	**Women (n = 23)**	**p value**
MG-H1	27.14 ± 11.24	27.56 ± 9.66	p > 0.5
GOLD	3.22 ± 0.10	3.21 ± 0.13	p > 0.1
MOLD	3.74 ± 0.23	3.64 ± 0.27	p > 0.05
	**Controls (n = 35)**		
	**Men (n = 15)**	**Women (n = 20)**	**p value**
MG-H1	30.87 ± 11.85	36.94 ± 20.59	p > 0.1
GOLD	3.39 ± 0.25	3.50 ± 0.25	p > 0.1
MOLD	3.89 ± 0.29	4.22 ± 0.80	p > 0.1

Table S2. Correlation analysis between concentrations of the examined compounds and age of study participants.

**Group**	**Compound**	**Age**
**Psoriasis Patients**	MG-H1	R = −0.25; p > 0.05
	GOLD	R = 0.00; p = 1
	MOLD	R = 0.5; p > 0.5
**Controls**	MG-H1	R = 0.21; p > 0.1
	GOLD	R = −0.28; p > 0.1
	MOLD	R = 0.37; p > 0.1

Table S3. Correlation analysis between concentrations of the examined compounds and indicators of disease severity (BSA, PASI), quality of life (DLQI), and duration of the disease.

**Compound**	**BSA**	**PASI**	**DLQI**	**Disease Duration**
MG-H1	R = −0.08; p > 0.5	R = 0.09; p > 0.5	R = 0.22; p > 0.05	R = 0.00; p = 1
GOLD	R = 0.17; p > 0.1	R = 0.13; p > 0.1	R = 0.11; p > 0.1	R = −0.17; p > 0.1
MOLD	R = 0.08; p > 0.5	R = 0.15; p > 0.1	R = 0.06; p > 0.5	R = −0.13; p > 0.1

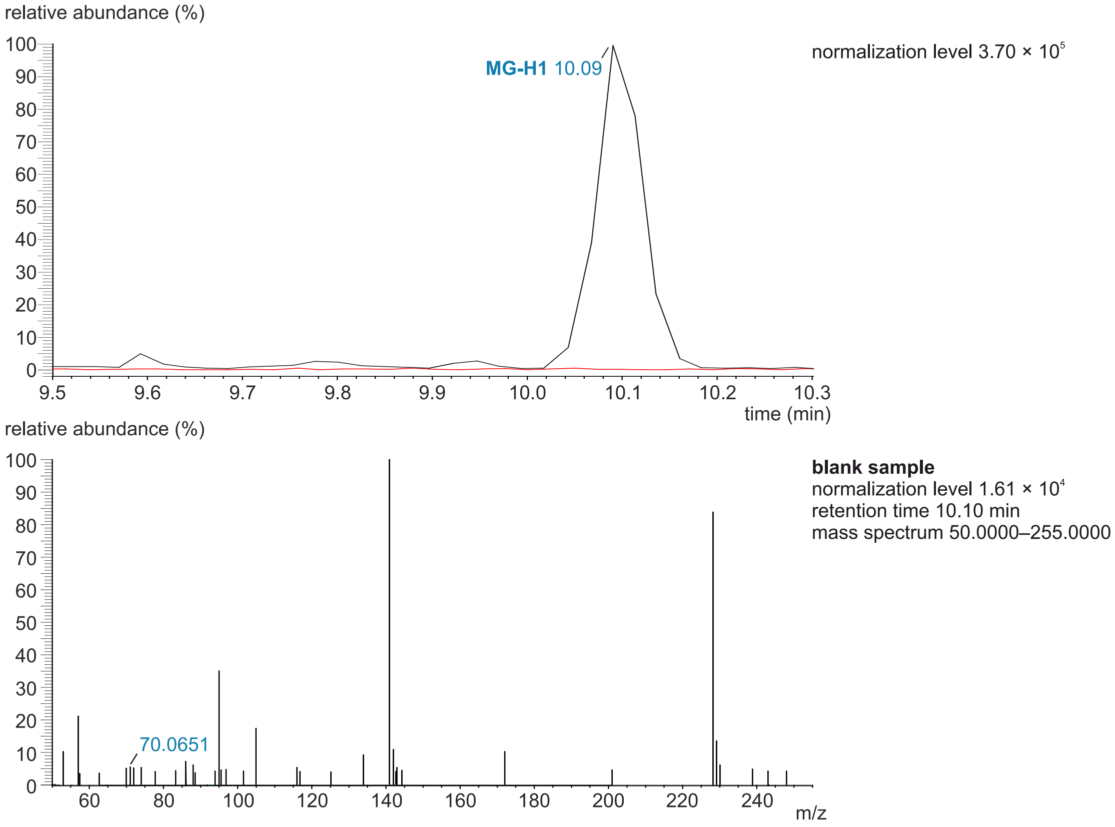

Figure S1.

Top: Fragments of the LC-Orbitrap-MS/MS chromatogram for the determination of MG-H1 (retention time 10.09 min) in serum extract sample (black) and acetonitrile (red) analyzed prior to the serum sample. The interference from the blank sample background is minimal.

Bottom: Mass spectrum for the signal recorded at 10.10 min in acetonitrile sample. This spectrum does not contain signals characteristic for MG-H1, namely: 114.065; 166.096 and 229.127 however low intensity peak 70.0652 is present.

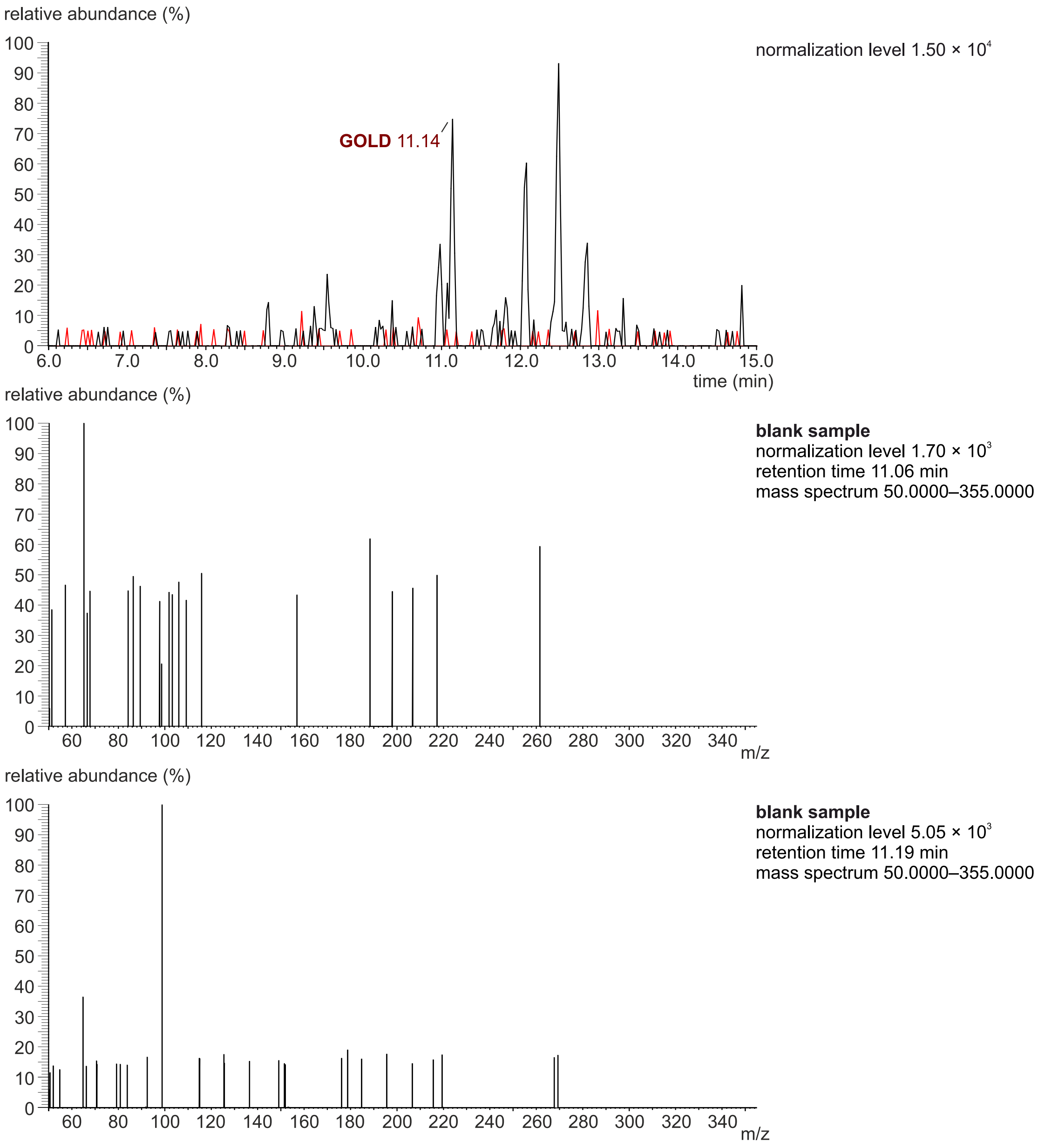

Figure S2.

Top: Fragment of the LC-Orbitrap-MS/MS chromatogram for the determination of GOLD (retention time 11.14 min) in serum extract sample (black) and in acetonitrile sample (red) analyzed before the serum sample.

Middle: Mass spectrum for the signal recorded at 11.06 min in the acetonitrile sample. This spectrum does not contain signals characteristic for GOLD, that is: 84.0806; 130.0853 and 153.1010.

Bottom: Mass spectrum for the signal recorded at 11.19 min in the acetonitrile sample. This spectrum does not contain signals characteristic for GOLD, that is: 84.0806; 130.0853 and 153.1010.

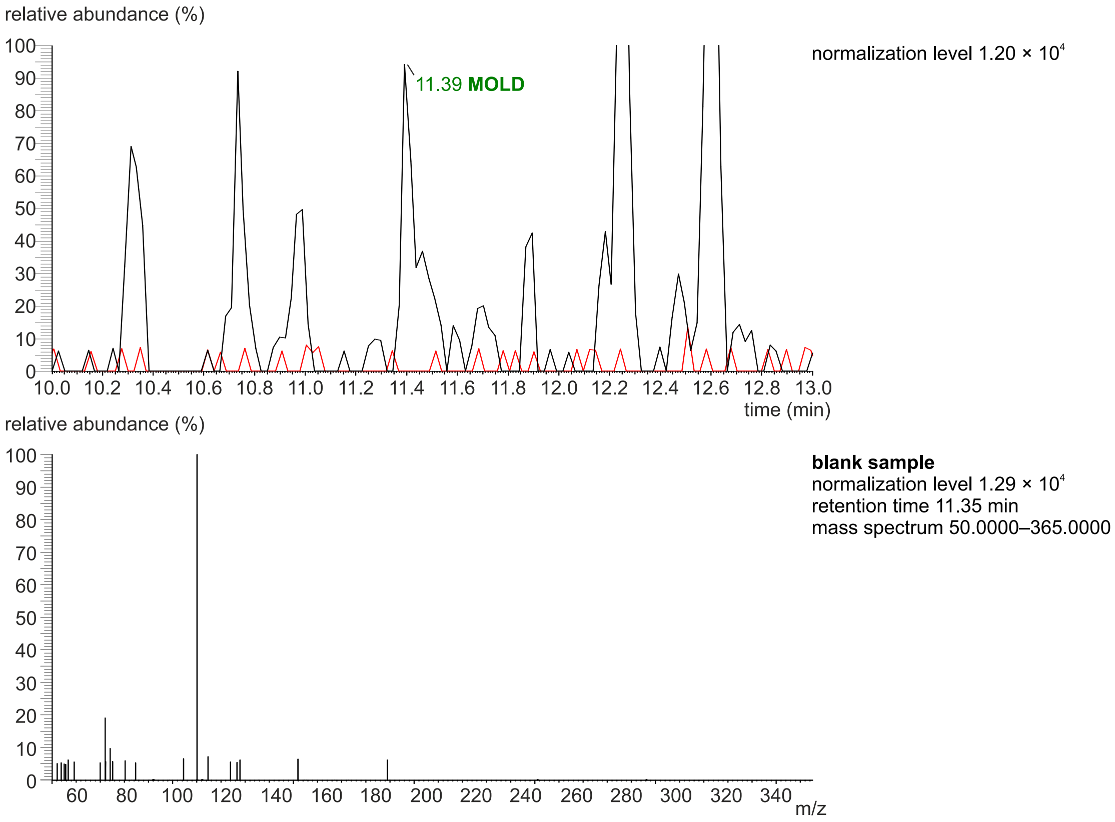

Figure S3.

Top: Fragment of the LC-Orbitrap-MS/MS chromatogram for the determination of MOLD (retention time 11.39 min) in control serum extract sample (black) and acetonitrile sample (red) analyzed before the serum sample.

Bottom: Mass spectrum for the signal recorded at 11.35 min in acetonitrile sample. This spectrum does not contain signals characteristic for MOLD, namely: 84.0806; 167.1163 and 251.1732.
